# Basic leucine zipper (*bZIP*) transcription factor genes and their responses to drought stress in ginseng, *Panax ginseng* C.A. Meyer

**DOI:** 10.1186/s12864-021-07624-z

**Published:** 2021-05-01

**Authors:** Hongjie Li, Jing Chen, Qi Zhao, Yilai Han, Li Li, Chunyu Sun, Kangyu Wang, Yanfang Wang, Mingzhu Zhao, Ping Chen, Jun Lei, Yi Wang, Meiping Zhang

**Affiliations:** 1 College of Life Science, Jilin Agricultural University, 2888 Xincheng Street, 130118 Changchun, Jilin China; 2 Jilin Engineering Research Center for Ginseng Genetic Resources Development and Utilization, Jilin Agricultural University, 2888 Xincheng Street, 130118 Changchun, Jilin China; 3 College of Chinese Medicinal Materials, Jilin Agricultural University, 2888 Xincheng Street, 130118 Changchun, Jilin China

**Keywords:** *Panax ginseng*, bZIP transcription factor, Phylogeny, Functional differentiation, Drought stress tolerance

## Abstract

**Background:**

Ginseng is an important medicinal herb in Asia and Northern America. The basic leucine zipper (*bZIP*) transcription factor genes play important roles in many biological processes and plant responses to abiotic and biotic stresses, such as drought stress. Nevertheless, the genes remain unknown in ginseng.

**Results:**

Here, we report 91 *bZIP* genes identified from ginseng, designated *PgbZIP* genes. These *PgbZIP* genes were alternatively spliced into 273 transcripts. Phylogenetic analysis grouped the *PgbZIP* genes into ten groups, including A, B, C, D, E, F, G, H, I and S. Gene Ontology (GO) categorized the *PgbZIP* genes into five functional subcategories, suggesting that they have diversified in functionality, even though their putative proteins share a number of conserved motifs. These 273 *PgbZIP* transcripts expressed differentially across 14 tissues, the roots of different ages and the roots of different genotypes. However, the transcripts of the genes expressed coordinately and were more likely to form a co-expression network. Furthermore, we studied the responses of the *PgbZIP* genes to drought stress in ginseng using a random selection of five *PgbZIP* genes, including *PgbZIP25*, *PgbZIP38*, *PgbZIP39*, *PgbZIP53* and *PgbZIP54*. The results showed that all five *PgbZIP* genes responded to drought stress in ginseng, indicating that the *PgbZIP* genes play important roles in ginseng responses to drought stress.

**Conclusions:**

These results provide knowledge and gene resources for deeper functional analysis of the *PgbZIP* genes and molecular tools for enhanced drought tolerance breeding in ginseng.

**Supplementary Information:**

The online version contains supplementary material available at 10.1186/s12864-021-07624-z.

## Background

Ginseng (*Panax ginseng* C.A. Meyer) is an important medicinal herb in Asia and Northern America. In China, ginseng has a long cultivation history and is mainly cultivated in Jilin Province where it is known as Jilin ginseng. Ginsenosides, present in most tissues of ginseng, are recognized as the most valuable active components of ginseng [1, 2]. Ginseng has a lot of benefits for human, such as relieving pain, improving brain function, and increasing anti-tumor activity [3–5]. However, ginseng is frequently suffering from different biotic and abiotic stresses, including, but not limited to, diseases, insect pests, drought, cold, heat, and daylight intensity, greatly threatening its production. Therefore, it is necessary to comprehensively investigate the genes involved in plant defense to the stresses in ginseng.

Transcription factors (TFs) have been shown to play a vital role in plant responses to various biotic or abiotic stresses. The basic leucine zipper (bZIP) transcription factor containing a conserved bZIP domain that is composed of 60–80 amino acids is known as one of the largest TF families [6, 7]. The conserved bZIP domain is composed of two important functional regions: the basic region and the leucine zipper region, linked by one hinge [8, 9]. The basic region usually contains an invariant N-x7-R/K motif (approximately 16 amino acids) and is responsible for both nuclear localization and DNA binding. The leucine zipper region mediates the homo- and/or hetero-dimerization as it contains a less conserved dimerization motif [10–13].

The *bZIP* genes have been documented to play a vital role in a number of biological processes, including plant tissue and organ differentiation and vascular development [14, 15], embryogenesis [16], and seed maturation [17]. Studies have also shown that the *bZIP* genes code key components in plant regulation of biotic and abiotic stresses, e.g., pathogens [18, 19], osmosis [20, 21], salinity [22, 23], cold [13, 24], and drought [25, 26]. It has been reported that *AtbZIP28* was activated by thermal stress, and then regulated the expressions of heat-responsive genes to protect plants from heat stress [27]. In rice, *OsbZIP23* and *OsbZIP72* were reported to attenuate drought stress by activating ABA signaling [28, 29]. Knockouting *SlbZIP1* and *SlAREB1* that belong to Group A of the *SlbZIP* gene family increased salt stress tolerance, while the over-expressions of *SlbZIP1* and *SlAREB1* decreased salt stress tolerance in tomato [30, 31].

The *bZIP* gene family has been analyzed in several plant species and shown to vary in size. For example, 75 *bZIP* genes were identified in Arabidopsis [10], 69 in tomato [32], and 89 in rice [7]. However, no research on the *bZIP* gene family has been reported yet in ginseng. The present study first identified the 91 *bZIP* genes from ginseng, which were designated *PgbZIP* genes. We then examined their conserved protein motifs, phylogeny, putative functionality, and expression characteristics and co-expression networks in different tissues, different year-old roots, and in the roots of different genotypes. Because drought stress restricts plant growth and development [33], influencing ginseng production, *PgbZIP* genes were further studied in response to drought stress in ginseng.

## Results

### Identification of *PgbZIP* genes

A total of 1,957 transcript sequences containing the bZIP domain were identified from Database A consisting of 248,993 transcripts. As the conserved domains of 1,684 of the 1,957 transcripts were incomplete or out of ORFs (open-reading frames), the remaining 273 transcripts that contain complete bZIP domains in their ORFs were identified as the *PgbZIP* gene transcripts for Jilin ginseng. These 273 transcripts had a sequence length of 210 to 3,651 bp, with an average length of 1,449 bp (Additional file 1: Table S1). Analysis showed that these 273 transcripts were alternatively spliced from 91 *PgbZIP* genes [34]. Of these 273 *PgbZIP* transcripts, 190 contained full-length ORFs that were derived from 62 *PgbZIP* genes. The full-length proteins encoded by the 190 *PgbZIP* transcripts contained amino acids varying from 46 (*PgbZIP84*) to 785 (*PgbZIP63-1*), with an average of 294 amino acids (Additional file 2: Table S2). In comparison, 45 (49 %) of the 91 Jilin ginseng *PgbZIP* genes identified in this study were orthologous to 111 (76 %) of the 146 Korean ginseng *PgbZIP* genes. The remaining 46 (51 %) of the Jilin ginseng *PgbZIP* genes were newly discovered or Jilin ginseng-specific (Additional file 3: Table S3; Additional file 4: Fig. S1).

### Phylogeny and conserved motifs of the *PgbZIP* gene family

**Fig. 1:**
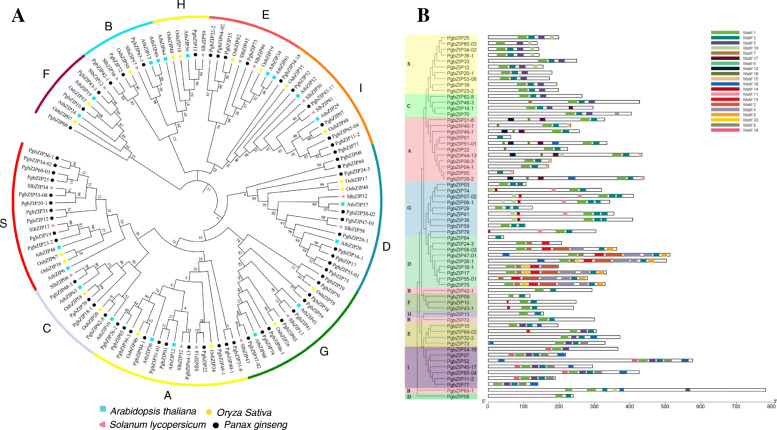
Phylogenetic relationship and conserved motifs of the *PgbZIP* proteins. **a** The NJ phylogenetic tree of the *PgbZIP* proteins constructed using the bZIP proteins of Arabidopsis, tomato and rice as the outgroups. **b** Conserved motifs of *PgbZIP* proteins according to their evolutionary relationship. The conserved motifs of the *PgbZIP* proteins are indicated by colored boxes. The capital letters indicate the groups of the *PgbZIP* gene family

The longest transcript for each of the 62 genes containing a full-length ORF was used to construct the NJ (neighbor-joining) phylogenetic tree of the *PgbZIP* gene family. Fifty-six *bZIP* genes that were identified from Arabidopsis (20 *AtbZIP* genes), rice (19 *OsbZIP* genes) and tomato (17 *SlbZIP* genes) (Additional file 5: Table S4) were used as outgroups. The 62 *PgbZIP* genes were clustered into ten clades, defined ten groups in this study, with the *AtbZIP, OsbZIP* and *SlbZIP* genes from Arabidopsis, rice and tomato (Fig. 1a). This result suggested that the *PgbZIP* gene family is an ancient gene family that originated before splitting between the monocot (rice) and dicot (Arabidopsis and tomato) plants. The *PgbZIP* gene family has the same number of groups as the *AtbZIP*, *OsbZIP* or *SlbZIP* gene family [8], but consists of more groups than the *bZIP* gene family of castor bean [35], cucumber [36] or sorghum [37]. Group A of the *PgbZIP* gene family has the largest number of *PgbZIP* genes, with 11 *PgbZIP* genes and Group H has only one *PgbZIP* gene (*PgbZIP13*). Similarly, we also constructed the MP (maximum parsimony) tree for the *PgbZIP* gene family (Additional file 6: Fig. S2). The MP tree was essentially the same as the NJ tree, with a difference from the NJ tree in grouping of only *PgbZIP08, PgbZIP09* and *PgbZIP84*, which was likely due to their low bootstrap confidences for both the NJ and MP trees.

Twenty conserved motifs were identified from the putative proteins encoded by the 62 *PgbZIP* genes that were spliced into transcripts with full-length ORFs (Additional file 2: Table S2). The distribution of these 20 conserved motifs in the 62 *PgbZIP* genes is shown in Fig. 1b. Motif 1, annotated as the bZIP domain, is presented in all 62 *PgbZIP* genes of the gene family. The putative proteins of most *PgbZIP* genes in a group of the gene family usually have a similar set of motifs (Fig. 1b). For instance, the putative proteins of most *PgbZIP* genes of Group A contains Motifs 3, 12, 15, 16 and 17; those of Group D harbor Motifs 2, 4, 6, 7, 8, 13 and 20; and those of Group G share Motif 11. Moreover, some groups of the *PgbZIP* gene family also share the same motif. For instance, Groups G and S both possess Motif 10, and Groups F and G are common in Motif 14. These results suggested the similarities of the *PgbZIP* genes in functionality.

### Functional differentiation of the *PgbZIP* gene family

**Fig. 2:**
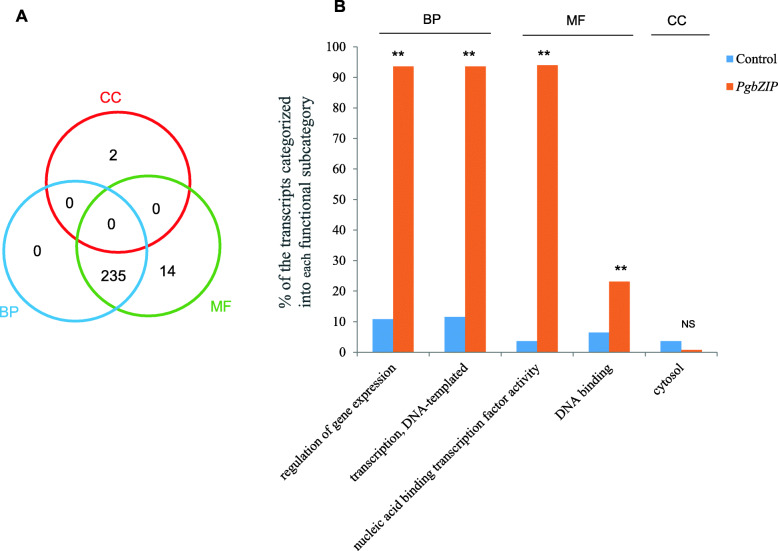
Functional categorization and GO term enrichment of the *PgbZIP* gene transcripts. **a** Venn diagram of numbers of the *PgbZIP* transcripts categorized into the biological process (BP) (235 transcripts), molecular function (MF) (249 transcripts) and cellular component (CC) (2 transcripts) categories. **b** Subcategories (Level 2) into which the *PgbZIP* transcripts are categorized and their enrichments. The GO terms of the transcripts expressed in 14 tissues of the four-year-old plant used for identification of the *PgbZIP* genes as the background control for the enrichment analysis. “**”, significant at *P* ≤ 0.01; NS, not significant at *P* ≤ 0.05

We examined the functional differentiation of the *PgbZIP* gene family by categorizing the 273 *PgbZIP* gene transcripts using Gene Ontology (GO). Two hundred fifty-one (91.9 %) of the 273 *PgbZIP* gene transcripts were annotated and categorized into all three primary categories, Biological Process (BP), Molecular Function (MF) and Cellular Component (CC) (Fig. 2a; Additional file 7: Table S5). BP contained 235 *PgbZIP* transcripts, MP contained 249 *PgbZIP* transcripts and CC had two *PgbZIP* transcripts (Fig. 2a). At Level 2, these 251 *PgbZIP* gene transcripts were categorized into five subcategories, including two BP subcategories (transcription DNA-templated and regulation of gene expression), two MF subcategories (nucleic acid binding transcription factor activity and DNA binding), and one CC subcategory (cytosol) (Fig. 2b). Of these 5 subcategories, all except cytosol were enriched in number of *PgbZIP* transcripts (*P* ≤ 0.01).

**Fig. 3:**
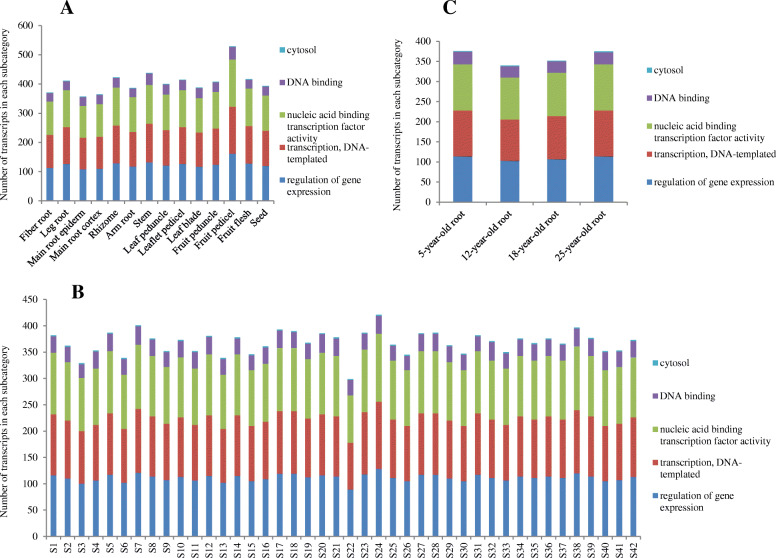
Variation of the functional categories of the *PgbZIP* transcripts. **a** Number variation of the *PgbZIP* transcripts categorized into a subcategory among 14 tissues of a 4-year-old plant. **b** Number variation of the *PgbZIP* transcripts categorized into a subcategory among the 4-year-old roots of 42 genotypes. **c** Number variation of the *PgbZIP* transcripts categorized into a subcategory among the roots of differently aged plants

The *PgbZIP* gene transcripts expressed in 14 tissues (Fig. 3a) of a four-year-old plant, the four-year-old roots of 42 genotypes (Fig. 3b), and the roots of four differently aged plants (Fig. 3c) were also categorized, respectively. These *PgbZIP* transcripts were also categorized into the above mentioned five subcategories, but the numbers of the *PgbZIP* gene transcripts categorized into the five subcategories varied substantially across tissues, genotypes and developmental stages. These results together demonstrated the functional differentiation of the *PgbZIP* gene family and also confirmed their functional consistency across tissues, genotypes and developmental stages.

### Expression characteristics of the *PgbZIP* gene transcripts

To characterize the expressions of the *PgbZIP* genes, the 273 *PgbZIP* transcripts were investigated in expression in different tissues, different year-old plant roots, and the four-year-old roots of different genotypes (Additional file 8: Table S6). The *PgbZIP* transcripts were used for this experiment because different transcripts spliced from one gene may have different biological functions [38]. The expressions of the *PgbZIP* transcripts varied dramatically, ranging from 0.0 TPM to 307.7 TPM, to 178.5 TPM, and to 169.9 TPM among different tissues, different year-old plant roots, and different genotypes, respectively.

**Fig. 4:**
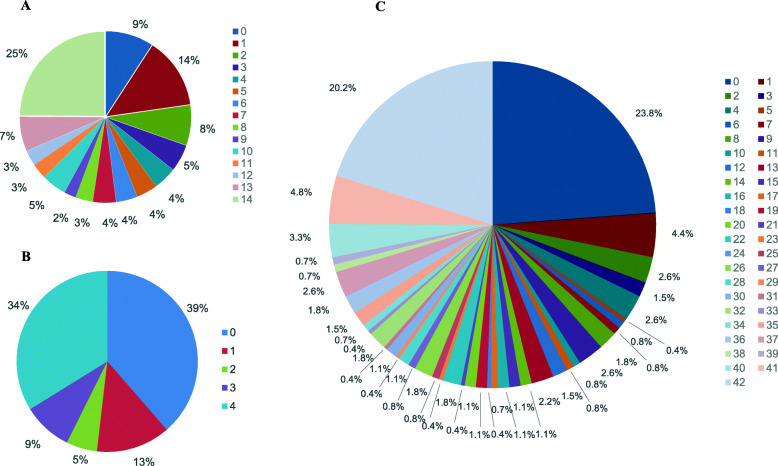
Numbers of the 273 *PgbZIP* gene transcripts expressing across tissues, the roots of differently aged plants, and genotypes. **a** Percentage of the *PgbZIP* gene transcripts expressing in different numbers of tissues. The number of tissues from 0 through 14 are indicated by different colorful squares. **b** Percentage of the *PgbZIP* gene transcripts expressing in the roots of differently aged plants from 0 through 4. **c** Percentage of the *PgbZIP* gene transcripts expressing in four-year-old roots of different numbers of genotypes from 0 through 42. “0” indicates the numbers of the 273 *PgbZIP* gene transcripts did not express in any of the 14 tissues of the plant, the root of any of the four differently aged plants analyzed or any of the 42 genotypes analyzed

For different tissues of a four-year-old plant, 248 (91 %) of the 273 *PgbZIP* transcripts expressed in at least one tissue (relative to the reference transcriptome), but most (77 %) expressed in two or more tissues. Sixty-eight (25 %) *PgbZIP* transcripts expressed in all 14 tissues and 37 *PgbZIP* transcripts (14 %) showed tissue-specific expressions (Fig. 4a). For different developmental stages, 167 (61 %) of the 273 *PgbZIP* transcripts expressed at least in one of 5-, 12-, 18- and 25-year-old plant roots, 93 (34 %) expressed at all four developmental stages, 35 (13 %) were developmental stage expression-specific, and 39 (14 %) expressed at two or three of the developmental stages (Fig. 4b). For the four-year-old roots of different genotypes, 208 (76 %) of the 273 *PgbZIP* transcripts expressed in at least one genotype, 55 (20 %) expressed in all 42 genotypes, 12 (4 %) were genotype expression-specific, and 141 (52 %) expressed in 2–41 genotypes (Fig. 4c). For individual tissues, root developmental stages or genotypes, from 40 − 60 % of the 273 *PgbZIP* transcripts expressed (Additional file 9: Fig. S3).

**Fig. 5:**
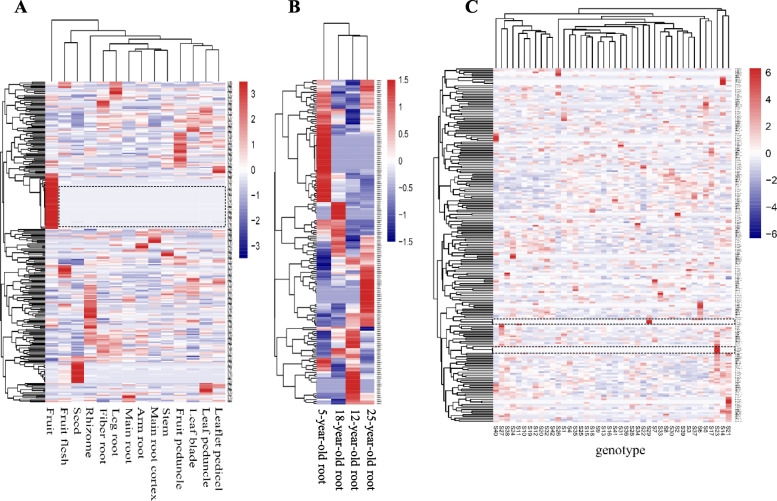
Expression heatmaps of the *PgbZIP* transcripts. **a** Heatmap of the *PgbZIP* transcripts constructed with their expressions in 14 tissues. **b** Heatmap of the *PgbZIP* transcripts constructed with their expressions in four different year-old roots. **c** Heatmap of the *PgbZIP* transcripts constructed with their expressions in the roots of 42 genotypes. The genes that were co-regulated are marked by dot-line boxes

Furthermore, gene expression heatmaps were constructed for the 273 *PgbZIP* transcripts that expressed in different tissues (Fig. 5a), different year-old plant roots (Fig. 5b) and the four-year-old roots of different genotypes (Fig. 5c) to estimate their co-regulations and expression patterns. The results showed that a number of the *PgbZIP* transcripts had identical expression patterns across the 14 tissues, four different developmental stages or 42 genotypes, suggesting that they were co-regulated.

### The co-expression network of the *PgbZIP* genes

**Fig. 6:**
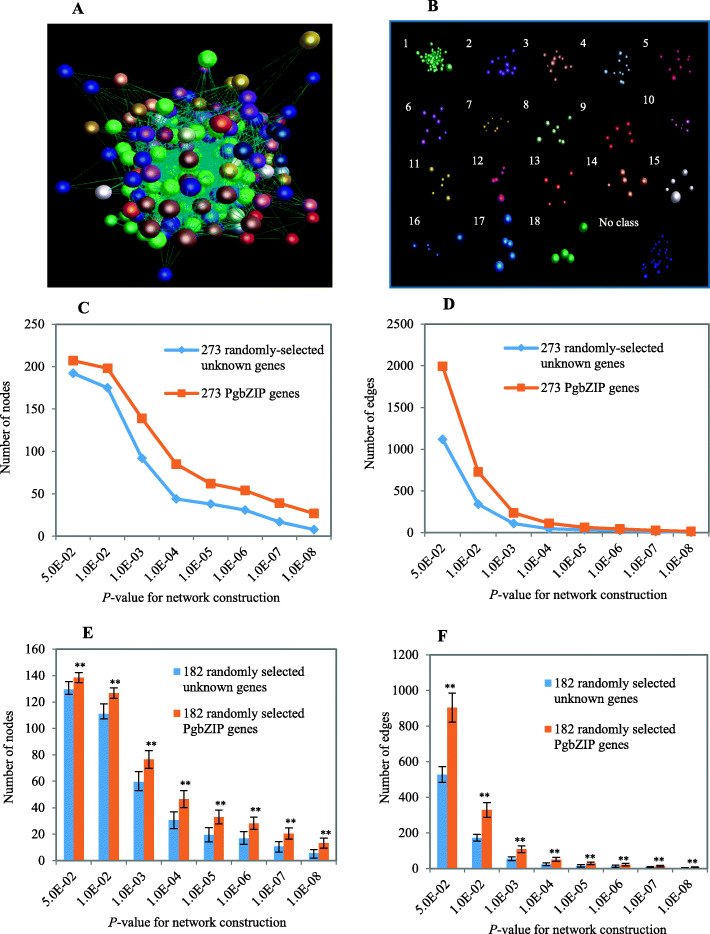
Network analysis of the *PgbZIP* transcripts expressed in the 4-year-old roots of 42 genotypes. **a** The co-expression network of the 273 *PgbZIP* transcripts constructed at *P* ≤ 5.0E-02. It consists of 208 nodes and 1,994 edges. **b** The 18 clusters of the network. **c** Tendency that *PgbZIP* transcripts form a network using the randomly-selected ginseng unknown transcripts as a control: variation in number of nodes. **d** Tendency that *PgbZIP* transcripts form a network using the randomly-selected ginseng unknown transcrips as a control: variation in number of edges. **e** Statistics of variation in number of nodes in the *PgbZIP* network. **f** Statistics of variation in number of edges in the *PgbZIP* network. “**”, significant at *P* ≤ 0.01; Error bar, the standard deviation for 20 bootstrap replications

To assess the potential functional relationships among different members of the *PgbZIP* gene family, the co-expression network of its 91 *PgbZIP* genes was constructed based on the expressions of their 273 transcripts in the four-year-old plant roots of 42 genotypes at a *P* ≤ 0.05 (Fig. 6). Two hundred seventy-three functionally unknown Jilin ginseng gene transcripts were randomly selected from Database A as the negative controls. Consequently, 208 of the 273 *PgbZIP* transcripts formed a co-expression network that was composed of 208 nodes, 1,994 edges (Fig. 6a), and 18 clusters (Fig. 6b). In comparison, the co-expression network of the *PgbZIP* transcripts was much more robust than that constructed from the 273 randomly-selected unknown ginseng transcripts at all significance levels from *P* ≤ 5.0E-02 through *P* ≤ 1.0E-08 (Fig. 6c,d). Statistical analysis confirmed the tendency that *PgbZIP* transcripts was more likely to form a co-expression network than the randomly-selected unknown ginseng transcripts (Fig. 6e,f). These results concluded that the members of the *PgbZIP* gene family were more likely to form a co-expression network, suggesting that they likely function correlatively.

### Response of the *PgbZIP* gene family to drought stress

**Fig. 7:**
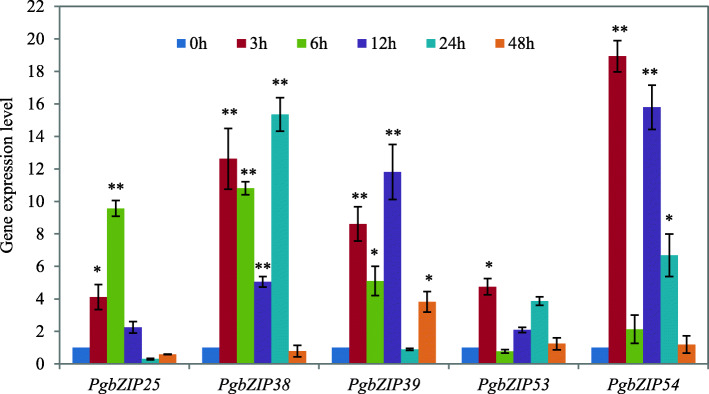
Expression levels of five *PgbZIP* genes randomly selected from 91 *PgbZIP* genes in ginseng seedlings treated with 20 % PEG-6000 after 0, 3, 6, 12, 24 and 48 h. The values are presented as the means of three replicates. “*”, *P* ≤ 0.05. “**”, *P* ≤ 0.01

To test whether the *PgbZIP* gene family functions in plant response to drought stresses, five *PgbZIP* genes, *PgbZIP25*, *PgbZIP38*, *PgbZIP39*, *PgbZIP53* and *PgbZIP54*, were randomly selected from the *PgbZIP* gene family and examined in plant response to drought stress. Ginseng seedlings were treated with 20 % (w/v) PEG-6000 that is widely used to drought-stress plants for 3, 6, 12, 24 and 48 h. The RWCs (relative water contents) of the seedlings treated with and without PEG-6000 were determined and compared. The RWCs of the seedlings treated with PEG-6000 for 24 h were significantly reduced, relative to the control seedlings not treated with PEG-6000. No significant difference in RWC was observed for the seedlings treated with PEG-6000 for other time points (Additional file 10: Fig. S4). Nevertheless, the expressions of all five *PgbZIP* genes studied were up-regulated in the seedlings treated with PEG-6000. Specifically, the expressions of *PgbZIP25*, *PgbZIP38*, *PgbZIP39*, *PgbZIP53* and *PgbZIP54* in the seedlings treated with PEG-6000 for 3 h were up-regulated by 4.1-, 12.6-, 8.6-, 4.7- and 18.9-fold, respectively, over those in the untreated seedlings (*P* ≤ 0.01). However, as the treatment time increased the expressions of these five genes varied differently. For instance, the expression of *PgbZIP25* reached the peak after the seedlings stressed with PEG-6000 for 6 h, while no significant different expression of the gene was observed in the treated seedlings from that in the control seedlings after stressed for 12 h, 24 and 48 h. *PgbZIP38* was continuously up-regulated in the seedlings treated by PEG-6000 for 3 h through 24 h, but returned to the expression level as in the control seedlings at 48 h. The expression of *PgbZIP53* in the seedlings stressed with PEG-6000 for 6 h through 48 h showed no significant difference from that in the control seedlings. *PgbZIP39* and *PgbZIP54* showed irregular expression variation in the seedlings stressed at different time points (Fig. 7).

## Discussion

We have identified 91 *PgbZIP* genes that expressed in a four-year-old Jilin ginseng plant. The size of the *PgbZIP* gene family is comparable to those identified in rice (89 *bZIP* genes) [7], *Brachypodium distachyon* (96) [9], barley (89) [12], and sorghum (92) [37], but larger than those identified in Arabidopsis (75) [10], castor bean (49) [35], cucumber (64) [36] and grapevine (55) [39], and smaller than those identified in maize (125) [8] and soybean (160) [13]. This result suggests that the *PgbZIP* gene family is an intermediate-sized transcription factor gene family. It seems independent of its genome size and perennial growth nature.

Phylogenetic analysis in the present study shows that the *PgbZIP* gene family is made up of 10 groups, which is the same as the *bZIP* gene families identified in Arabidopsis, rice, tomato and maize. This result suggests that the *PgbZIP* gene family has a similar evolutionary trajectory to those of Arabidopsis, rice, tomato and maize. Notably, all ten groups of the *PgbZIP* gene family are grouped with the *bZIP* genes of Arabidopsis, tomato and rice, implying that the *PgbZIP* gene family has existed before splitting between monocots (rice) and dicots (ginseng, Arabidopsis, tomato). The clustering of the *PgbZIP* gene family and its significant sharing of conserved motifs with those of Arabidopsis, rice and tomato indicate that they have a common ancestor.

It has been reported that the *bZIP* genes are involved in a variety of biological processes, including drought/osmotic stress response [9, 36, 37], growth and development and cell elongation [40, 41], organ and tissue differentiation [42], and seed storage protein gene regulation [17]. This study categorizes the *PgbZIP* genes into five subcategories that belong to all three primary categories. This result suggests that the functionality of the *PgbZIP* gene family has been substantially differentiated. However, the functional differentiation of the gene family was far smaller than those of the *PgRLK* gene family (23 subcategories) [43] and the *PgNBS* gene family (36 subcategories) [44] identified in Jilin ginseng. Our result indicates that the *PgbZIP* gene family mainly functions in DNA-templated transcription, regulation of gene expression, and nucleic acid binding transcription factor activity.

The *PgbZIP* gene family actively expresses in all tissues, at all developmental stages and in all genotypes examined in this study, but only approximately 50 % of the genes in the family expressed in a tissue, at a developmental stage and in a genotype. Although the expression activities of the genes in the *PgbZIP* gene family vary dramatically across tissues, at different developmental stages and in different genotypes, most of them expressed in multiple tissues and only a small portion are tissue-, developmental stage- or genotype-specific. Expression heatmap analysis reveals that co-regulation of the *PgbZIP* gene expressions exists across tissues, developmental stages and genotypes, but the co-regulation is observed only for a limited number of the *PgbZIP* genes. The tendency of co-expression network formation for the majority of the *PgbZIP* genes indicates the functional correlation of the *PgbZIP* genes and also their functional differentiation.

Previous studies showed that the *bZIP* genes isolated from mung bean, adzuki bean, Arabidopsis, wheat, rice, and *Tamarix hispida* were involved in plant response to drought and salt stresses [45–49]. This study shows that all five *PgbZIP* genes, *PgbZIP39*, *PgbZIP25*, *PgbZIP38*, *PgbZIP53* and *PgbZIP54*, randomly selected from the A, S, G, S and I groups of the *PgbZIP* gene family, respectively, responded to the drought stress stimulated by PEG-6000. This result confirms that the *PgbZIP* gene family also plays a role in plant response to drought stress. Nevertheless, additional research will be needed to further investigate the tolerance of the *PgbZIP* genes to drought stress and their underlying molecular mechanisms.

## Conclusions

Ninety-one *PgbZIP* genes were identified from Jilin ginseng and systematically analyzed in phylogeny, conservation, functional differentiation, expression, and network interaction. The *PgbZIP* gene family is an ancient gene family and has substantially differentiated in functionality. The expressions of the *PgbZIP* genes varied spatially, temporally and across genotypes, but they were more likely to form a co-expression network, suggesting their functional correlation. It is observed that the *PgbZIP* gene family is involved in plant response to drought stress in ginseng. Together, the results of this study conclude that the *PgbZIP* gene family consists of at least 91 gene members, its functionality has diverged but its members remain functionally correlated at a substantial degree, and it likely plays a significant role in plant response to drought stress.

## Methods

### Databases

The Jilin Ginseng Transcriptome Databases developed from 14 tissues of a four-year-old plant (Database A), the roots of four different year-old plants (Database B) [50], and the four-year-old roots of 42 genotypes collected from Jilin province, China, defined from S1 to S42 (Database C) (Additional file 11: Table S7) [44, 51] were used for this study. All the samples for the databases were collected at the fruiting stage of the plants.

### Identification of the *bZIP* gene family in ginseng

Three steps were conducted to identify the *bZIP* genes from Jilin ginseng. First, the local Hidden Markov Model (HMM) search was carried out with the bZIP domain HMM profiles, including PF07716, PF00170, PF03131, and PF012498 (http://pfam.sanger.ac.uk/), using the HMMER3.0 software (HMMER: http://hmmer.wustl.edu/). Then, BLAST search was performed at a threshold of 1.0E-06 to identify putative *bZIP* genes from Database A. Third, all putative *bZIP* genes were subjected to filtration by the Online Conserved Domain Search. The resulting *bZIP* genes were identified as *PgbZIP* genes that were defined from *PgbZIP01* through *PgbZIP91* and whose transcripts were indicated with suffix, such as “_1, _2, and so on” (Additional file 1: Table S1).

### Comparison of the *PgbZIP* genes between Jilin ginseng and Korean ginseng

To have an overview of the Jilin ginseng *PgbZIP* genes with those identified from the genome database of Korean ginseng, we compared these two sets of *PgbZIP* genes. The sequences of the Korean ginseng *PgbZIP* genes were downloaded from the Korean Ginseng Genome Database (http://ginsengdb.snu.ac.kr/index.php) [52] and aligned with the Jilin ginseng *PgbZIP* genes identified from the Jilin Ginseng Transcriptome Database (see above). The sequences with an identity of ≥ 95 % and an alignment length of ≥ 240 bp (the bZIP domain maximum length) were identified as the orthologous genes of the Jilin and Korean ginsengs in this study [53].

### Phylogenetic analysis of the *PgbZIP* gene family in Jilin ginseng

The Jilin ginseng *PgbZIP* genes were translated into putative proteins and those having complete ORFs were selected as representatives for phylogenetic analysis of the *PgbZIP* gene family, with one longest bZIP protein sequence per gene. The full-length protein sequences of the *bZIP* genes of *Arabidopsis thaliana* (Arabidopsis*)*, *Solanum lycopersicum* (tomato) and *Oryza sativa* (rice) were downloaded from the Plant TF Database (PlantTFDB, v3.0) [54], the Sol Genomics Network (SGN; http://solgenomics.net/) and the Rice Genome Annotation Project RGAP (http://rice.plantbiology.msu.edu/) [32], respectively, and used as outgroups. Then, the *PgbZIP*-coding proteins of ginseng, along with those of Arabidopsis, rice and tomato, were subjected to multiple sequence alignment. Finally, the phylogenetic tree of the *PgbZIP* gene family was constructed using MEGA version 7.0 (http://www.megasoftware.net), with the Neighbor-Joining (NJ) and Maximum Parsimony (MP) algorithm, respectively and 1,000 replications.

### Conserved motifs of the *PgbZIP* genes

The ORFs of the full-length *PgbZIP* transcripts were first identified using the online ORF Finder at NCBI (http://www.ncbi.nlm.nih.gov/gorf/gorf.html). Then, the conserved motifs of the *PgbZIP* proteins were searched using the Motif Elicitation Tool (version 4.9.1, http://meme.sdsc.edu/meme/cgi-bin/meme.cgi) [55] with a maximum number of 20. The minimum and maximum lengths of the conserved motifs were 10 and 50 amino acids, respectively, and other parameters were used as default [56].

### Functional categorization of the *PgbZIP* transcripts

The 273 *PgbZIP* transcripts were submitted to Blast2GO (Version 4.1.5) to perform GO analysis and categorization according to their GO terms. The number of transcripts categorized into each functional subcategory was subjected to enrichment analysis [57] using the GO functional categorization of 1,000 unknown ginseng gene transcripts randomly selected from Database A [50] as controls. The difference between the observed number of *PgbZIP* transcripts and the expected number of randomly-selected unknown ginseng transcripts categorized into each functional subcategory (Level 2) were examined by Chi-square test.

### Expressions and network analysis of the *PgbZIP* transcripts

The expressions of the 273 *PgbZIP* transcripts were extracted from Databases A [50], B [50] and C [44], respectively. The R programming language was used to construct the expression heatmaps of the 273 *PgbZIP* transcripts and the BioLayout Express^3D^ software (Version 3.2) was used to construct their co-expression networks [58].

### Plant growth and drought stress

The seeds of Jilin ginseng, Damaya, were grown in the pots containing nutritional soil for 4 weeks. Then, the four-week-old seedlings were treated with 20 % PEG-6000 (polyethylene glycol 6000) to simulate drought stress. PEG-6000 has been widely used to mimic drought stress for study of plant response to drought stress [59–61]. After treated with PEG-6000 for 3 h, 6 h, 12 h 24 and 48 h, the seedlings were harvested and weighed immediately (fresh weight). The seedlings were then placed in a vial containing distilled water at 4℃ for 24 h, and the saturated weight of the seedlings was measured. Third, these seedlings were dried at 70℃ for 72 h, and their dry weights were measured. Finally, the relative water contents (RWCs) of the control and drought-stressed seedlings were measured using the following formula: [(fresh weight - dry weight) / (saturated weight - dry weight)] x 100 [59].

### RNA isolation and analysis by quantitative real‐time PCR

After treated with PEG-6000 for 0 h, 3 h, 6 h, 12 h 24 and 48 h, the total RNAs of seedlings were isolated by TRIzol reagent (Biotake, Beijing, China) and the first-strand cDNAs were synthesized using the PrimeScript™ RT reagent Kit with gDNA Eraser (TaKaRa, Tokyo, Japan). Ubiquinol-cytochrome C reductase (*QCR*) gene of ginseng was selected as the reference gene [62]. Five *PgbZIP* genes, *PgbZIP25*, *PgbZIP38*, *PgbZIP39*, *PgbZIP53* and *PgbZIP54*, were randomly selected from the 91 *PgbZIP* genes and the primers specific for these genes were designed and synthesized (Additional file 12: Table S8). qRT-PCR was conducted using an Applied Biosystems 7500 Real Time PCR System (Thermo Fisher Scientific,Waltham, USA) and SYBR Premix Ex Taq™ II (TaKaRa, Tokyo, Japan). The PCR conditions were 30 s at 95℃, and followed by 40 cycles of 5 s at 95℃, 34 s at 60℃, and one cycle of 15 s at 95℃, 60 s at 60℃, and finally, 15 s at 95℃. Three biological replicates were conducted and the gene relative expression was calculated using the 2^−ΔΔ*C*T^ formula.

## Supplementary information

Additional file 1: **Table S1.** The *PgbZIP* genes identified in this study and their transcript sequences.

Additional file 2: **Table S2.** The 62 *PgbZIP* genes that were spliced into transacripts having full-length ORFs and their putative protein sequences.

Additional file 3: **Table S3.** Sequence alignment of the Jilin ginseng *PgbZIP* genes to the Korean Ginseng Genome Database.

Additional file 4: **Fig. S1.** Comparison of the number of *PgbZIP* genes between the Korean Ginseng Genome Database and the Jilin Ginseng Transcriptome Database.

Additional file 5: **Table S4.** The published *bZIP* genes of Arabidopsis, rice and tomato used as outgroups for phylogenetic analysis of the *PgbZIP* gene family.

Additional file 6: **Fig. S2.** Phylogenetic tree of PgbZIP proteins constructed with the MP algorithm using the bZIP proteins of Arabidopsis, tomato and rice as the outgroups.

Additional file 7: **Table S5.** Annotation and GO categorization of the 273 *PgbZIP* transcripts.

Additional file 8: **Table S6.** Expressions of the 273 *PgbZIP* transcripts in the four-year-old roots of 42 genotypes, 14 tissues of a four-year-old plant and 4 different year-old roots.

Additional file 9: **Fig. S3.** Percentage of the 273 *PgbZIP* transcripts expressed in different tissues (**a**), in the roots of differently aged plants (**b**), in the four-year-old roots of different genotypes (**c**).

Additional file 10: **Fig. S4. **Relative water content (RWC) of ginseng seedlings subjected to PEG stress. The control plants were maintained under normal water irrigation conditions. The values are presented as the means of three replicates. “*”, *P* ≤ 0.05.

Additional file 11: **Table S7.** Plant materials used for this study.

Additional file 12: **Table S8.** Primers used for qRT-PCR analysis.

## Data Availability

The data used for this study are available at Sequence Read Archive (SRA) of National Center for Biotechnology Information (NCBI), BioProject PRJNA302556; and at Gene Expression Omnibus (GEO) of NCBI, SRP066368 and SRR13131364 - SRR13131405. The plant materials are available through corresponding authors, upon request.

## Abbreviations

bZIP: basic leucine zipper.
*PgbZIP*: *Panax ginseng* bZIP
TF: transcription factor
ORF: open reading frame
MEME: multiple EM for motif elicitation
NJ: neighbor-joining
MP: maximum parsimony
GO: gene ontology
MF: molecular function
BP: biological process
CC: cellular component
HMM: Hidden Markov Model
PEG-6000: polyethylene glycol 6000
RWC: relative water content
