# Supplementary material for: Basic leucine zipper (bZIP) transcription factor genes and their responses to drought stress in ginseng, Panax ginseng C.A. Meyer
Source: BMC Genomics. 2021 May 1;22:316. doi: 10.1186/s12864-021-07624-z (PMC8088647; doi:10.1186/s12864-021-07624-z)
Supplement: Supplementary file 4 — Fig. S1. Comparison of the number of PgbZIP genes between the Korean Ginseng Genome Database and the Jilin Ginseng Transcriptome Database. [file 12864_2021_7624_MOESM4_ESM.pptx]

## Slide 1
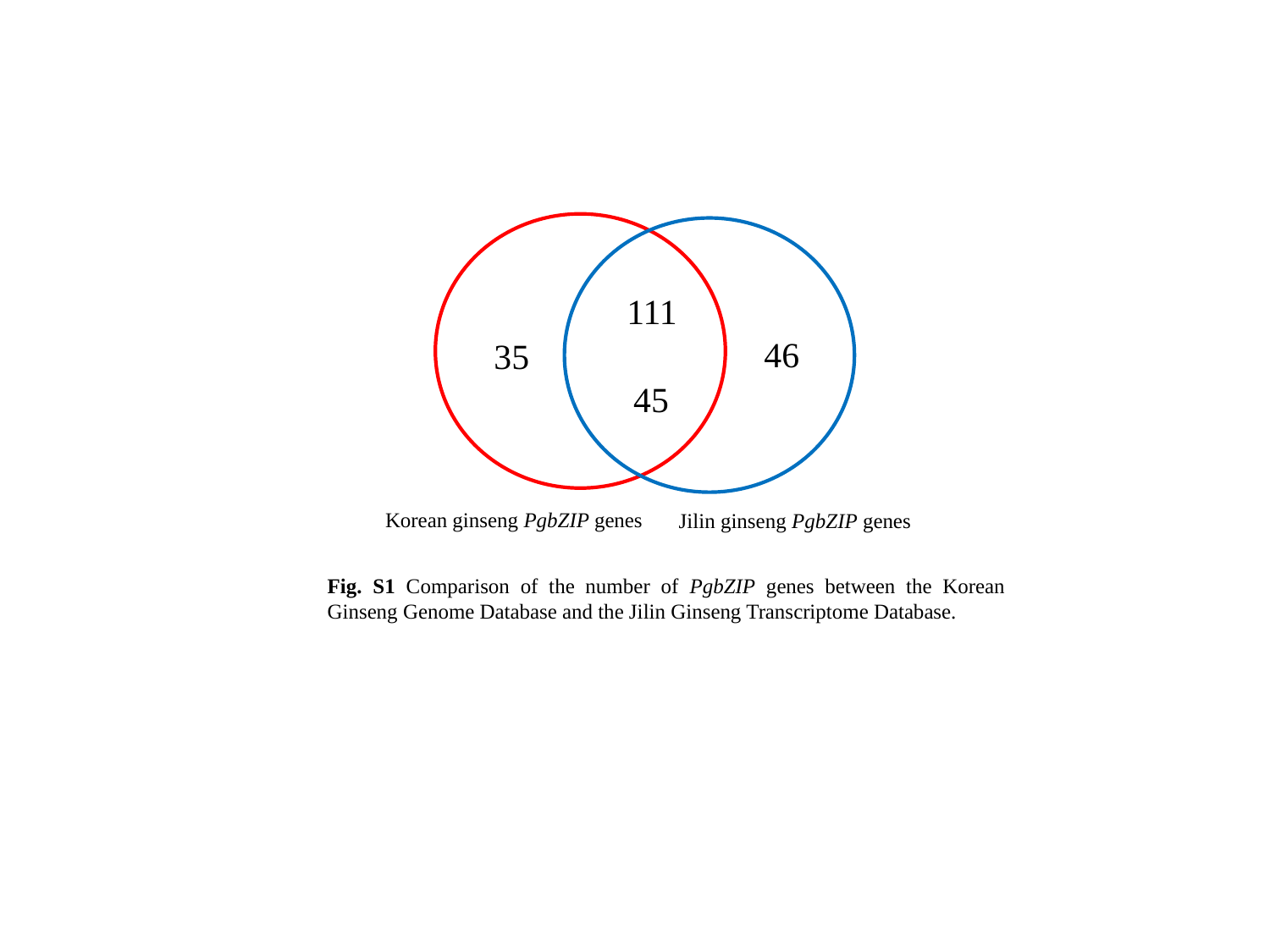

111
46
35
45
Korean ginseng PgbZIP genes
Jilin ginseng PgbZIP genes
Fig. S1 Comparison of the number of PgbZIP genes between the Korean Ginseng Genome Database and the Jilin Ginseng Transcriptome Database.
