# Supplementary material for: Basic leucine zipper (bZIP) transcription factor genes and their responses to drought stress in ginseng, Panax ginseng C.A. Meyer
Source: BMC Genomics. 2021 May 1;22:316. doi: 10.1186/s12864-021-07624-z (PMC8088647; doi:10.1186/s12864-021-07624-z)
Supplement: Supplementary file 6 — Fig. S2. Phylogenetic tree of PgbZIP proteins constructed with the MP algorithm using the bZIP proteins of Arabidopsis, tomato and rice as the outgroups. [file 12864_2021_7624_MOESM6_ESM.pptx]

## Slide 1
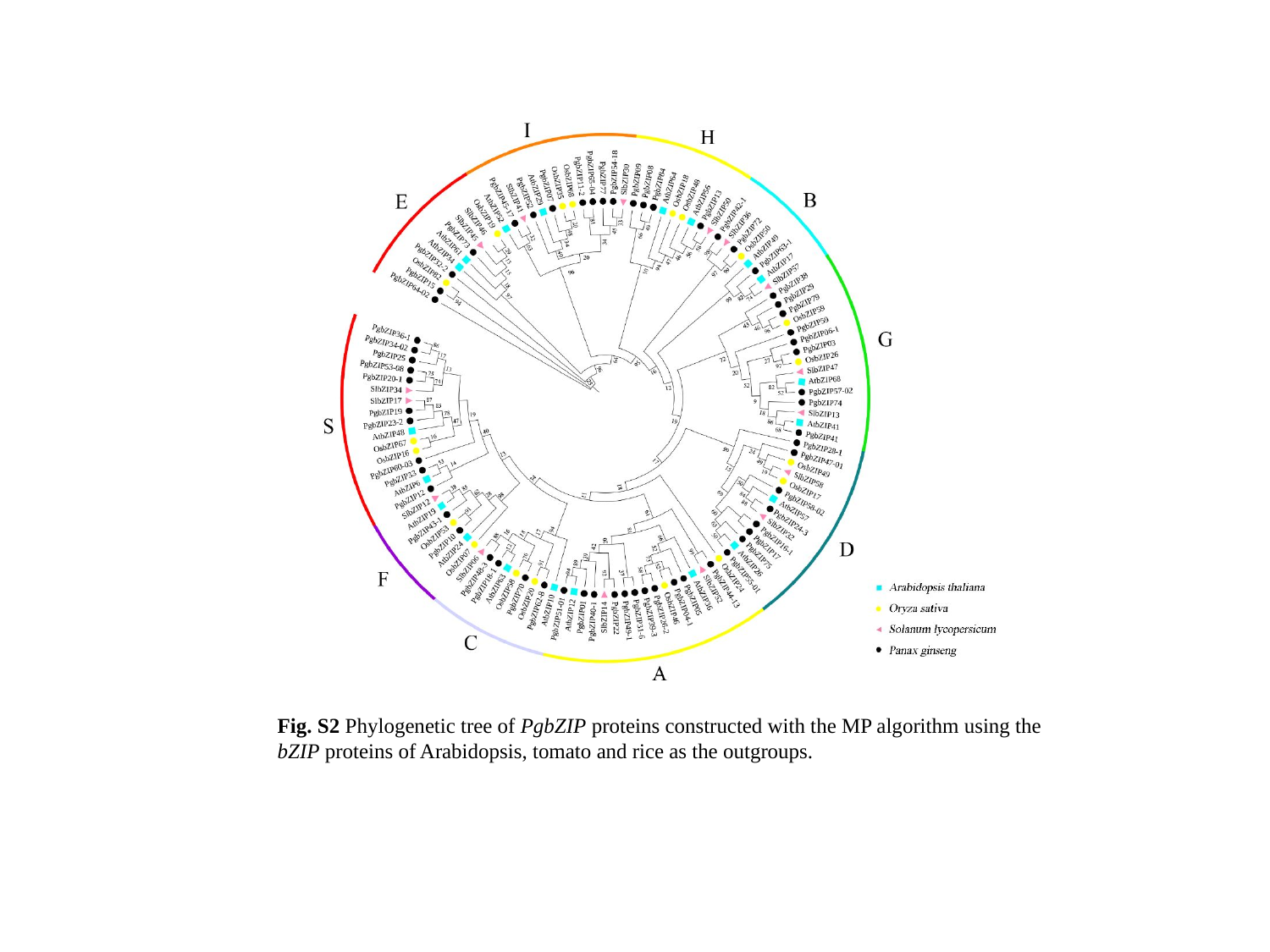

Fig. S2 Phylogenetic tree of PgbZIP proteins constructed with the MP algorithm using the bZIP proteins of Arabidopsis, tomato and rice as the outgroups.
