# Supplementary material for: Basic leucine zipper (bZIP) transcription factor genes and their responses to drought stress in ginseng, Panax ginseng C.A. Meyer
Source: BMC Genomics. 2021 May 1;22:316. doi: 10.1186/s12864-021-07624-z (PMC8088647; doi:10.1186/s12864-021-07624-z)
Supplement: Supplementary file 9 — Fig. S3. Percentage of the 273 PgbZIP transcripts expressed in different tissues (a), in the roots of differently aged plants (b), in the four-year-old roots of different genotypes (c). [file 12864_2021_7624_MOESM9_ESM.pptx]

## Slide 1
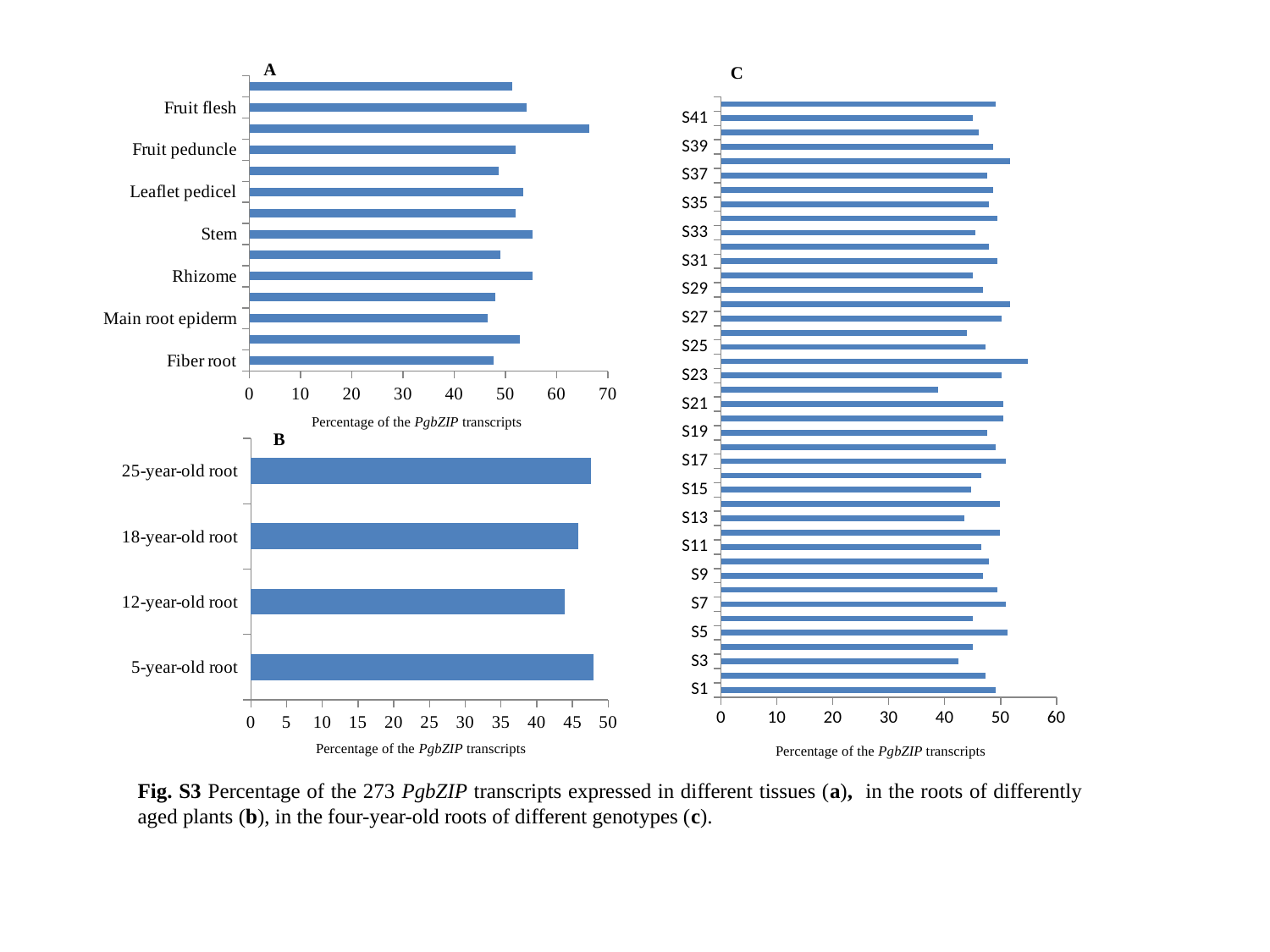

A
C
### Chart
| Category | |
|---|---|
| S1 | 49.08424908424908 |
| S2 | 47.25274725274725 |
| S3 | 42.49084249084249 |
| S4 | 45.05494505494506 |
| S5 | 51.28205128205128 |
| S6 | 45.05494505494506 |
| S7 | 50.91575091575091 |
| S8 | 49.45054945054945 |
| S9 | 46.88644688644688 |
| S10 | 47.985347985347985 |
| S11 | 46.52014652014652 |
| S12 | 49.81684981684982 |
| S13 | 43.58974358974359 |
| S14 | 49.81684981684982 |
| S15 | 44.68864468864469 |
| S16 | 46.52014652014652 |
| S17 | 50.91575091575091 |
| S18 | 49.08424908424908 |
| S19 | 47.61904761904761 |
| S20 | 50.54945054945055 |
| S21 | 50.54945054945055 |
| S22 | 38.82783882783883 |
| S23 | 50.18315018315018 |
| S24 | 54.94505494505495 |
| S25 | 47.25274725274725 |
| S26 | 43.956043956043956 |
| S27 | 50.18315018315018 |
| S28 | 51.64835164835166 |
| S29 | 46.88644688644688 |
| S30 | 45.05494505494506 |
| S31 | 49.45054945054945 |
| S32 | 47.985347985347985 |
| S33 | 45.42124542124542 |
| S34 | 49.45054945054945 |
| S35 | 47.985347985347985 |
| S36 | 48.717948717948715 |
| S37 | 47.61904761904761 |
| S38 | 51.64835164835166 |
| S39 | 48.717948717948715 |
| S40 | 46.15384615384615 |
| S41 | 45.05494505494506 |
| S42 | 49.08424908424908 |
### Chart
| Category | |
|---|---|
| Fiber root | 47.61904761904761 |
| Leg root | 52.74725274725275 |
| Main root epiderm | 46.52014652014652 |
| Main root cortex | 47.985347985347985 |
| Rhizome | 55.311355311355314 |
| Arm root | 49.08424908424908 |
| Stem | 55.311355311355314 |
| Leaf peduncle | 52.01465201465202 |
| Leaflet pedicel | 53.47985347985348 |
| Leaf blade | 48.717948717948715 |
| Fruit peduncle | 52.01465201465202 |
| Fruit pedicel | 66.30036630036629 |
| Fruit flesh | 54.21245421245421 |
| Seed | 51.28205128205128 |Percentage of the PgbZIP transcripts
B
### Chart
| Category | |
|---|---|
| 5-year-old root | 47.985347985347985 |
| 12-year-old root | 43.956043956043956 |
| 18-year-old root | 45.78754578754579 |
| 25-year-old root | 47.61904761904761 |Percentage of the PgbZIP transcripts
Percentage of the PgbZIP transcripts
Fig. S3 Percentage of the 273 PgbZIP transcripts expressed in different tissues (a), in the roots of differently aged plants (b), in the four-year-old roots of different genotypes (c).
