# Supplementary material for: Basic leucine zipper (bZIP) transcription factor genes and their responses to drought stress in ginseng, Panax ginseng C.A. Meyer
Source: BMC Genomics. 2021 May 1;22:316. doi: 10.1186/s12864-021-07624-z (PMC8088647; doi:10.1186/s12864-021-07624-z)
Supplement: Supplementary file 10 — Fig. S4. Relative water content (RWC) of ginseng seedlings subjected to PEG stress. The control plants were maintained under normal water irrigation conditions. The values are presented as the means of three replicates. “*”, P ≤ 0.05. [file 12864_2021_7624_MOESM10_ESM.pptx]

## Slide 1
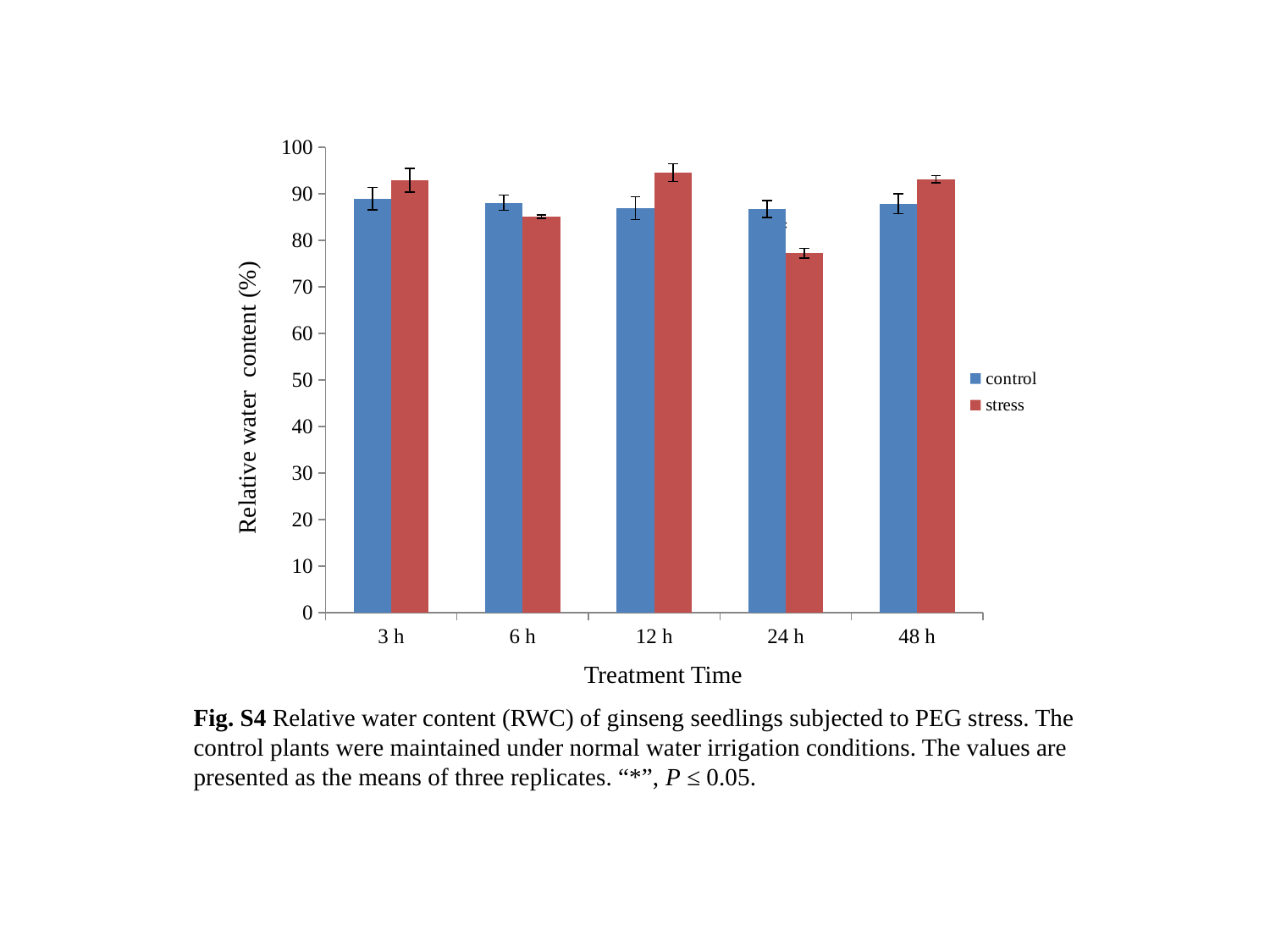

### Chart
| Category | control | stress |
|---|---|---|
| 3 h | 88.95724158882054 | 92.97364205621085 |
| 6 h | 88.1022868859992 | 85.11219824392983 |
| 12 h | 86.90986103179135 | 94.58190751952267 |
| 24 h | 86.71459182968134 | 77.25401572415306 |
| 48 h | 87.87834972045499 | 93.16908581614463 | *
Relative water content (%)
Treatment Time
Fig. S4 Relative water content (RWC) of ginseng seedlings subjected to PEG stress. The control plants were maintained under normal water irrigation conditions. The values are presented as the means of three replicates. “*”, P ≤ 0.05.
